# Innate Viral Receptor Signaling Determines Type 1 Diabetes Onset

**DOI:** 10.3389/fendo.2017.00249

**Published:** 2017-09-26

**Authors:** Zachary J. Morse, Marc S. Horwitz

**Affiliations:** ^1^Department of Microbiology and Immunology, University of British Columbia, Vancouver, BC, Canada

**Keywords:** type 1 diabetes, autoimmunity, innate immunity, toll-like receptors, RIG-I-like receptors, MDA5, type I and III interferon, coxsackievirus B

## Abstract

Heritable susceptibility of the autoimmune disorder, type 1 diabetes (T1D), only partially equates for the incidence of the disease. Significant evidence attributes several environmental stressors, such as vitamin D deficiency, gut microbiome, dietary antigens, and most notably virus infections in triggering the onset of T1D in these genetically susceptible individuals. Extensive epidemiological and clinical studies have provided credibility to this causal relationship. Infection by the enterovirus, coxsackievirus B, has been closely associated with onset of T1D and is considered a significant etiological agent for disease induction. Recognition of viral antigens *via* innate pathogen-recognition receptors induce inflammatory events which contribute to autoreactivity of pancreatic self-antigens and ultimately the destruction of insulin-secreting beta cells. The activation of these specific innate pathways and expression of inflammatory molecules, including type I and III interferon, prime the immune system to elicit either a protective regulatory response or a diabetogenic effector response. Therefore, sensing of viral antigens by retinoic acid-inducible gene I-like receptors and toll-like receptors may be detrimental to inducing autoreactivity initiated by viral stress and resulting in T1D.

## Introduction

Characterized by the destruction of the insulin-secreting beta cells of the pancreas and subsequent loss of blood glucose regulation, type 1 diabetes (T1D) is an autoimmune disorder whose onset is triggered by a combination of both genetic and environmental factors. Virus infections, vitamin D deficiency, dietary antigens, and disruption in the gut microbiota all have been implicated in eliciting T1D development in genetically susceptible individuals (1–4). Significant evidence suggests a strong causal association between genes involved in host–virus interactions and susceptibility to T1D. Using genome-wide association studies (GWAS), single nucleotide polymorphisms (SNPs) and gene variants conferring risk for T1D have been identified in multiple sites including the *interferon induced with helicase C domain 1* (*IFIH1*), *HLA class II, CTLA-4, insulin*, and *PTPN22* genes (5–7). The precise mechanisms leading to a loss of self-tolerance experienced in T1D are not adequately understood. Virus-mediated activation of T1D has been proposed to be caused by several different processes including direct islet infection, increased exposure to self-antigens which may have been previously sequestered, bystander activation, and molecular mimicry (8, 9).

Natural drift of genetic predisposition cannot adequately explain why the incidence of T1D has increased approximately 1.8% annually from 2002 to 2012 worldwide (10, 11). The concordance rate for T1D among monozygotic twins is about 35% by age 60, signifying significant contributions from environmental factors ultimately leads to the onset of autoimmunity (12). Indeed, epidemiological evidence indicates a link between virus infections and development of T1D as well as multiple other autoimmune disorders, including multiple sclerosis (MS), systemic lupus erythematosus (SLE), and rheumatoid arthritis (RA). Studies have demonstrated geographical and seasonal differences, as well as disease outbreaks, correlate with increased incidence of T1D (11, 13–17).

Upon virus infection, initial innate sensing likely primes genetically susceptible or protected individuals for an effector or regulatory immunological response, respectively (18). Therefore, signaling from pattern-recognition receptors (PRRs) that identify pathogen-associated molecular patterns (PAMPs) associated with certain viruses could determine whether infection will promote T1D induction. The production of interferon (IFN) from this PRR–PAMP interaction is a prominent immunological response for defense of virus infections. All three types of IFN, type I (IFN-α, -β, -ε, -κ, and -ω), type II (IFN-γ), and type III (IFN-λ1, -2, -3, and -4), stimulate the production of pro-inflammatory molecules from the interferon-stimulated genes (ISGs) to induce a strong antiviral state to prevent spreading of the infection to surrounding cells and also to establish an adaptive immune response (19, 20). Accordingly, alterations in signaling stemming from PRR activation represent the foundational mechanisms leading to T1D development by producing an IFN signature which is conducive for autoimmunity.

## Innate Viral Receptors

Genome-wide association studies indicate heritable differences in viral receptors and their related genes influence T1D susceptibility. Functional diversity of innate PRRs due to genetic variants may push the immune homeostasis toward an imbalance between pathogen hypersusceptibility and autoimmunity. In conjunction with an inherent variation, several different viruses have been implicated in causing inappropriate responses leading to T1D (4, 21). Among these viral candidates, enteroviruses such as coxsackievirus B (CVB) have been the most notable etiological agent attributed to T1D (22–24).

Dependent on the signals received from PRRs, innate immune cells including dendritic cells (DCs) macrophages, monocytes, natural killer cells and innate lymphoid cells can contribute to establishing either an effector inflammatory response or a more tolerogenic response by secreting cytokines, chemokines, and through priming of naïve T cells. While cross-reactivity of lymphocytes due to homology between viral and endogenous antigens and have been proposed in the establishment of T1D, non-specific immune stimulation causing persistent and low-grade inflammation are more likely underlaying the cause of pathogen-induced triggering of autoimmunity (25). The scale of an immune response is reliant on tightly regulated activation and inhibitory signals which may tip into an exaggerated or improper response causing the loss of self-tolerance (26).

**Figure 1:**
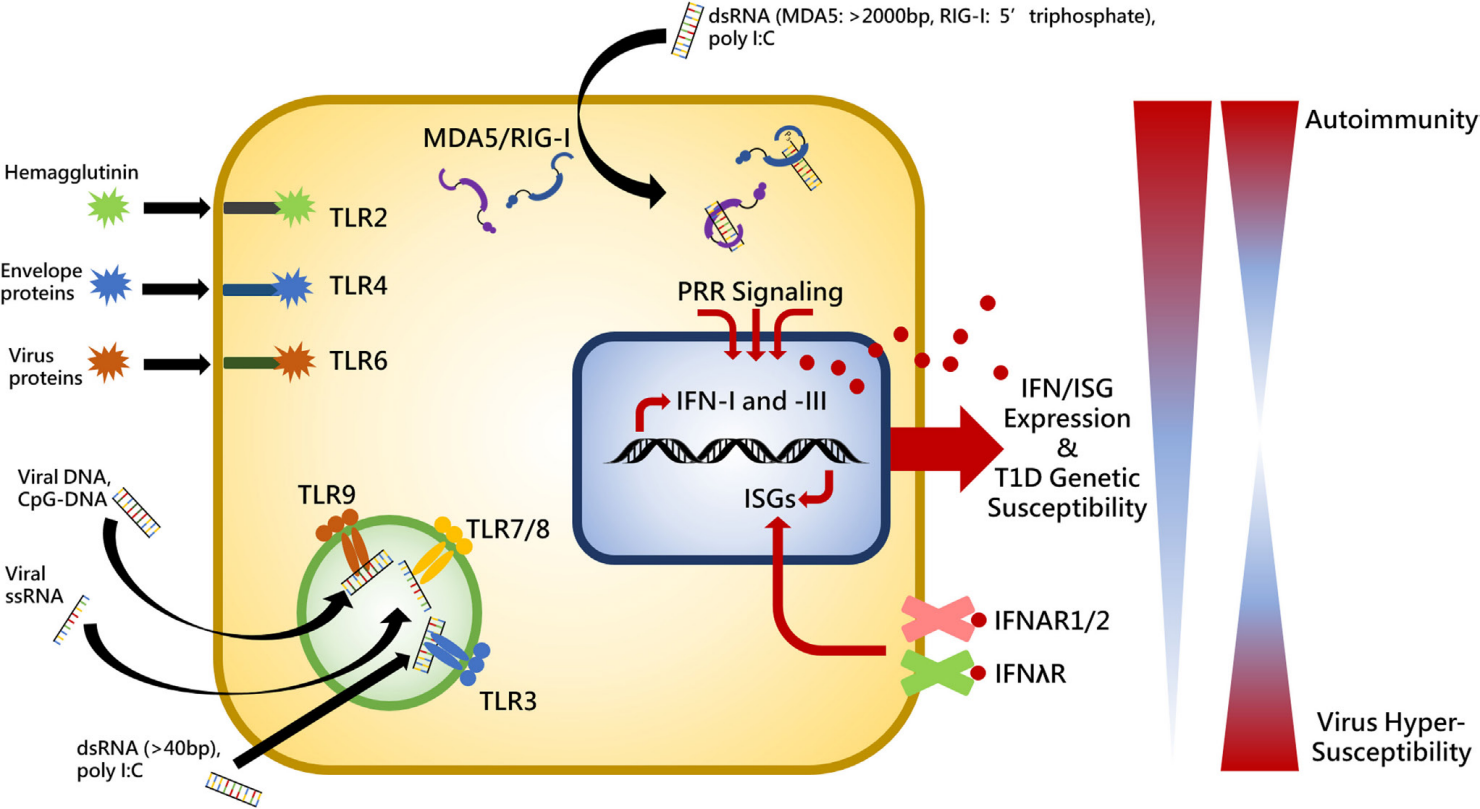
Summary of toll-like receptor (TLR)- and RIG-I-like receptors virus-associated ligands and the relationship between interferon (IFN) expression, genetic susceptibility, and autoimmunity. Upon ligand binding, cytosolic MDA5 and RIG-I receptors induce activation of the adaptor molecule, VISA (also called MAVS, IPS1, and CARDIF), endosomally located toll-like receptor 3 (TLR3) recruits TRIF (also known as TICAM), and TLR2, -4, -6, -7, -8, and -9 interact with myeloid differentiation primary response protein 88 (MYD88) in order to provide IFN expression stimulation in the cell nucleus. IFN induces expression of various interferon-stimulated genes (ISGs) which perform positive feedback on IFN genes. IFN is released from the cell to establish an antiviral state in surrounding cells and act in an autocrine and paracrine manner by binding to its cell surface receptors, IFNAR1/2 and IFNλR. Individuals exhibiting heightened genetic susceptibility to type I diabetes (T1D) can have increased basal and pathogen-elicited expression of IFN causing the immune system to skew toward a self-reactive state. Conversely, significantly diminished IFN expression would render a host unable to mount a proper response to virus infection. Thus balance of receptor stimulation between autoimmunity and virus hypersusceptibility is tightly regulated and pathogenic stimuli which exacerbates inflammation in genetically susceptible individuals may result in loss of tolerance.

Innate immunity and PRRs represent the first line of defense to coordinate the immune system for pathogen clearance and sets the stage for ensuing cellular and molecular pathway activation. The initial inflammatory state established with innate recognition of viral products induces beta cell damage and is then followed by apoptotic events and an effector T lymphocyte response killing the beta cells. Therefore, placing emphasis on the PRRs is critical for understanding the pathogenesis of autoimmune diabetes. There are three primary families of PRRs involved in detecting viral products: toll-like receptors (TLRs), retinoic acid-inducible gene I (RIG-I)-like receptors (RLRs), and nucleotide oligomerization domain-like receptors (27). Summarized in Figure 1, this review will focus on the contribution of RLRs and TLRs to T1D following engagement with their respective viral PAMPs.

## RLRs in T1D

The RLR family consists of RIG-I, melanoma differentiation-associated protein 5 (MDA5), and Laboratory of Genetics and Physiology 2 (LGP2), which are cytosolic receptors that recognize pieces of viral RNA from picornaviruses, flaviviruses, and paramyxoviruses (28). RLRs primarily bind viral replication intermediates [i.e., double-stranded RNA (dsRNA)] in infected cells and promote recruitment of transcription factors and adaptor molecules to restrict virus replication and prevent spread to other cells. Among a diverse range of effects, activation of MDA5 and RIG-I induce a potent type I and III IFN expression which go on to stimulate antiviral gene expression and increase antigen presentation (29, 30). LGP2 can bind short pieces of dsRNA and acts as a negative regulator for both RIG-I and MDA5; however, it lacks N-terminal caspase activation and recruitment domains necessary for signaling (31).

Expressed from the *IFIH1* gene, MDA5 is a cytosolic helicase which binds long viral dsRNA to induce a type I and III IFN response (18, 32). MDA5 has been identified as an important part of the host immune response to CVB and is necessary for preventing early replication of the virus and potentiating tissue damage (33). Various SNPs in the *IFIH1* gene have been found to confer either greater or reduced susceptibility for the onset of T1D (7). These SNPs likely alter the expression and activation of MDA5 when challenged with pathogenic stress. The A946T (rs1990760) mutation in *IFIH1* has been implicated in the development of multiple autoimmune diseases, including T1D, SLE, and MS (34, 35). Individuals which exhibit loss-of-function SNPs on even a single allele in RLR genes including E627* and I923V in MDA5 generally benefit from protection to T1D (35, 36). Hyperexpression or constitutive activation of MDA5 by mutagenesis has been shown to promote development of type I interferonopathies including SLE and Aicardi-Goutières syndrome (37–39). Diabetic patients which are heterozygous for the A946T SNP have a more robust ISG expression and immune response to CVB challenge when compared to healthy controls, potentially signifying an enhanced ability to promote IFN and ISG signal transduction during infection (40, 41). Accordingly, mutations in *IFIH1* causing gain-of-function are associated with hyperexpression of both IFN-I and -III (32, 39). Gorman et al. recently found that mice homozygous for the 946T variant as well as mice simultaneously exhibiting two *IFHI1* risk alleles (843R and 946T) have increased basal activation of *IFIH1*-related genes, enhanced protection from encephalomyocarditis virus infection, increased incidence of autoimmunity, and are inherently more sensitive to self RNA ligands (42). These mutations conferring T1D risk may be altering the homeostatic intensity of inflammatory molecule expression and/or the kinetics of target binding and activation—causing ligands to produce more potent or prolonged IFN responses. For example, the E627* mutation in MDA5 causes loss of a portion of the protein’s C-terminal region and consequently forfeiture of dsRNA ligand binding (36). The A946T risk variant is also associated with heightened sensitivity to IFN-α in SLE patients so this mutation may allow receptors to become more easily activated (43). This evidence supports the notion that pathogen-mediated T1D is likely similar to the described type I interferonopathy disorders.

Our lab has previously exhibited the importance of MDA5 signaling by demonstrating reduced expression of the receptor can be protective for T1D. Non-obese diabetic (NOD) mice which were heterozygous for a null *IFIH1* allele (MDA5^+/−^) and expressed roughly half as much MDA5 as wild-type NOD mice were shown to have decreased incidence of spontaneous disease (18). More importantly, upon CVB4 infection, these heterozygous mice were completely protected from diabetes onset while about 50% of homozygous NOD mice carrying a full complement of *IFIH1* developed T1D within 7 days of infection. MDA5 knockout mice were also completely protected from spontaneous T1D onset; however, they were highly susceptible to virus. Compared to homozygous mice, the MDA5^+/−^ mice displayed a specific type I IFN response characterized by a large spike in IFN-β occurring three days post-infection. It appears this particular IFN signature provides a succinct signal from IFN-β that is sufficient to clear the virus without inducing autoimmunity. Furthermore, MDA5^+/−^ mice had decreased CD4^+^ and CD8^+^ effector T cells as well as a robust CD4^+^CD25^+^Foxp3^+^ regulatory T cells (T_REG_) response that suppressed IFN-γ-producing CD4^+^ T cells, thereby preventing T1D.

## TLRs in T1D

Toll-like receptors are broadly expressed PRRs in both immune and non-immune cells which detect microbial- and viral-associated PAMPs (44). Upon recognition of pathogenic and/or foreign material, TLRs influence a number of immunologic mechanisms including activation and maturation of antigen-presenting cells (APCs), antibody production, downregulating T_REG_ responses, and inducing a pro-inflammatory environment through secretion of various cytokines and chemokines (45). Each of the TLRs may be stimulated with endogenous DNA or RNA antigens produced during cell death that may be a result of virus infection (46). However, those specifically recognizing viral-associated ligands: toll-like receptor 3 (TLR3), TLR7, TLR8, and TLR9 (and to a lesser extent TLR2, TLR4, and TLR6), have all been implicated in having a role in the diabetogenic potential of certain viruses (45, 47).

### Toll-Like Receptor 3

Binding short pieces of dsRNA, TLR3 is an endosomal receptor heavily expressed in classical DCs and a variety of epithelial cells (47). Unlike all other TLRs, TLR3 is MYD88-independent and instead utilizes the adaptor molecule TRIF for signal transduction following activation (44). The dsRNA mimetic polyinosinic:polycytidylic (poly I:C) is recognized by TLR3 and has been shown in various mouse studies to either protect or induce and increase severity of T1D depending on dose and administration (48–50). NOD mice deficient for TLR3 have high mortality from CVB4 infections and the few that survive develop T1D (51). However, in some instances, TLR3-KO NOD mice can show less severe insulitis as well as some reduced susceptibility to T1D induction following CVB4 infection, but experience no difference in spontaneous disease development (52). TLR3 signaling within resident macrophages is critical for antiviral host defense to CVB4 as well as altering marginal zone B cell composition in NOD mice (50, 51). This indicates that enhanced TLR3 activation may participate in T1D development as a result of virus infection. Certain polymorphisms in the *TLR3* gene have shown to be associated with increased risk of T1D and more aggressive pathology (rs3775291 and rs13126816) while others impart protection (rs5743313 and rs11721827) (53).

### Toll-Like Receptors 7 and 8

Expressed in the endosome, TLR7 and TLR8 recognize single-stranded RNA (ssRNA) while TLR9 is typically activated by binding unmethylated CpG DNA ligands from DNA viruses and microbial pathogens (54). TLR9-KO NOD mice have significantly lower rates of spontaneous diabetes, reduced activation of diabetogenic CD8^+^ cytotoxic T cells (CTLs), and elevated expression of the immunosuppressive marker CD73, particularly on T_REG_ cells (55–57). Thus, activation of TLR9 induces a less tolerogenic immunological state that contributes to the pathogenesis and acceleration of T1D.

Using rat insulin promoter mice expressing lymphocytic choriomeningitis virus glycoprotein (LCMV-GP), researchers have shown that LCMV infection produced IFN-α *via* stimulation of TLR3 and TLR7; this in turn increased the expression of MHC class I molecules in the insulin-secreting beta cells of the pancreas (58). This mechanism, where TLR-mediated expression of IFN-α upregulates MHC-I in the islets, was shown to be vital for the diabetogenic potential of LCMV and subsequent progression toward an overt autoreactive response. LCMV-GP-specific CTLs in the pancreas were unable to cause disease without hyperexpression of MHC-I (58). Stimulation of TLR7 in conjunction with CD40 activation of DCs can induce diabetogenic CTLs in the pancreatic lymph nodes of NOD mice to promote onset of autoimmunity (59). Even the repeated topical administration of a TLR7 agonist, imiquimod, is sufficient to promote T1D development while inhibition using IRS661 can significantly decrease onset (59). TLR7 signaling in plasmacytoid DCs (pDCs) primes B and T cell activation *via* IFN-I secretion in rotavirus infections; however, inhibition of TLR7 is able to block this process from occurring and prevent acceleration of T1D following infection (60). The role of TLR7 and TLR8 in promoting autoimmunity has also been indicated in CVB3-induced self-reactivity toward myocardial tissue (61).

### Environmental Inducers of TLRs

Previously, therapeutics for T1D prevention and treatment in the past have been primarily aimed at modifying or suppressing the adaptive immunity. Today, a shift in perspective of clinical methodology points toward targeting innate components to tolerize early pathogen-stimulated mechanisms as an effective strategy. Bednar and colleagues demonstrated that a TLR4-agonist monoclonal antibody, TLR4–MD-2, was able to halt and reverse fulminant T1D by inducing APC tolerance to pathogen in NOD mice (62). TLR4 is typically activated by lipopolysaccharides and other microbial products; however, envelope proteins from viruses including CVB can also stimulate its activation (63). Although it is uncertain whether CVB interaction with TLR4 is involved in T1D.

The natural route of enteroviral infection is through the gut, where the biodiversity of bacteria, viruses, fungi, and other microorganisms are significant mediators of immune homeostasis and autoimmunity (64). Accordingly, stimulation of the innate immunity and signaling on the mucosal surfaces from environmental pathogens could be detrimental to T1D onset. All TLRs other than TLR3 use the adaptor molecule MYD88 for signal transduction. Deletion of MyD88 in specific pathogen-free NOD mice confers resistance to diabetes (65). T1D resistance through loss of MYD88 is attributed to disruption in the gut flora since these MYD88-KO mice develop autoimmunity when housed in germ-free facilities (65). A “balanced signal hypothesis” has been suggested where microbiota-derived stimulation of TLR4 signaling through the adaptor molecule, TRIF, provides a tolerogenic effect on T1D pathogenesis, while TLR2 signaling promotes diabetogenesis (66). Commensal virus communities likely contribute similarly to innate stimulation through PRR recognition of viral ligands. A recent study has determined the intestinal virome is significantly altered prior to onset of autoimmunity in T1D-susceptible children (67). The overall diversity of the gut virome is reduced preceding disease development and certain viruses, such as *Circoviridae* and various bacteriophages, are significantly associated with either negative or positive T1D risk (67). This signifies a complex host–microbiome–virome relationship contributes to T1D and further studies are necessary to understand how these interactions alter disease and inflammation to skew genetically susceptible individuals toward either a protective or disease-causing state.

## T1D Displays Interferonopathy-Like Qualities

### Type I IFN

Pattern-recognition receptor activation and signaling remain the predominant inducer for IFN signatures that can protect as well as portend onset of not only T1D, but are also typical of rheumatic disorders such as SLE and RA (68, 69). The synergistic effects of type I and III IFNs are significant mediators for the adaptive immune system that promote lymphocyte maturation and mediate antigen presentation (19, 70). Accordingly, the IFN expression elicited by PRR activation is essential to autoimmune development. As such, it has been proposed that virus infections including CVB may be inducing localized interferonopathy-like characteristics within the islet microenvironment to trigger autoreactivity (71). Islets from patients recently experiencing onset of T1D exhibit heightened expression of certain ISGs in the islet and peri-islet regions in a manner which is similar to islets infected with virus (72). Knocking out the type I IFN receptor (IFNAR) in the T1D-susceptible rat strain, LEW.1WR1, protects from T1D, reduces insulitis, and delays onset following poly I:C or virus challenge (73). Originating with PRR stimulation, aberrant activation of pDCs and genetic mutations in the IFN signaling pathway likely contribute to the IFN signature evident in T1D induction (74).

Transient upregulation of type I IFN can be seen in genetically predisposed children preceding the seroconversion of T1D-related autoantibodies (75). Nearly all cells produce and respond to type I IFN; however, pDCs secrete a considerable amount of systemic IFN-α. Indeed, the secretion of IFN-α through TLR7- and TLR9-stimulated pDCs in the PLN of NOD mice is critical for onset of T1D (76). Blocking IFN-α signaling through IFNAR1 of young NOD mice (2–3 weeks old) significantly delays onset and incidence of diabetes as well as promote secretion of immunoregulatory cytokines, IL-4 and IL-10, in splenic CD4^+^ T cells (76). Treating human islet cells with IFN-α *in vitro* triggers endoplasmic reticulum stress which disrupts insulin production by hindering the conversion of proinsulin to insulin signifying a potential mechanism by which IFN-α may be prompting development of T1D (77). Using a neutralizing antibody against IFN-α or using a specific agonist for S1PR1, an immune regulatory receptor which mediates IFN-α autoamplification, protects T1D onset in a *Rip*-LCMV mouse model by limiting the infiltration of autoreactive T cells into the islets and by inducing expression of tolerogenic receptor genes, such as *Pdcd1, Lag3, Ctla4, Tigit*, and *Btla* (78). This immunomodulation is able to prevent the autoreactive T cells from harming the insulin-secreting beta cells thus preserving the glucoregulatory function of the pancreas. Accordingly, the progression from prediabetes to full-onset disease requires signaling from IFN-α.

The transcription factor, interferon regulatory factor 7 (IRF7), is constitutively expressed in pDCs and is expressed in most other cells upon IFNAR activation (79). IRF7 is involved in signal transduction from MYD88-dependant endosomal TLRs (TLR7, TLR8, and TLR9) as well as RLRs to trigger IFN gene expression. A study by Hienig et al. used rat tissues to elucidate the IRF7-driven inflammatory network (IDIN) to relate that genetic mapping with known viral response genes and disease GWAS (80). It was determined that an rs9585056 SNP (on chromosome 13q32), located in the orthologous human genes controlling IDIN, was significantly associated with susceptibility to T1D and promoted expression of the IRF7-driven signaling network. Similar to gain-of-function mutations in *IFIH1*, this type of genetic predisposition would cause vigorous antiviral engagement resulting in an IFN and immune response which may be more pathogenic than the actual virus.

### Type III IFN

While all nucleated cells respond to type I IFN, the type III IFN receptor (IFNλR) is primarily only expressed on pDCs and epithelial cells including pancreatic islet cells. Type III IFNs bind to the IFNλR consisting of dimer of IFNLR1 and IL10R2 domains. There is significant overlap in signaling pathways and activation between IFN-III and IFN-I; however, non-redundant roles for IFN-III in host antiviral responses exist (70, 81). Islets from humans exhibiting a protective *IFHI1* rs1990760 (946A/T) polymorphism produce an increased IFN-III response following CVB3 infection, likely through IRF-1 signaling, when compared to individuals with a risk-associated genotype (946T/T) (32). It is uncertain whether this additional expression of IFN-λ has protective qualities or whether it is simply a compensatory mechanism for lower IFN-I signaling from MDA5. However, IFN-λ-treated DCs are able to promote the specific proliferation of T_REG_ cells *in vitro* and IFN-λ treatment has been exhibited to improve pathology of RA in mice by reducing inflammatory neutrophils (82, 83). Collectively, this signifies IFN-III may be contributing to diabetes pathogenesis and should be further studied.

## Timing is Important for the Viral Etiology of T1D

### Acute versus Persistent Infections

**Figure 2:**
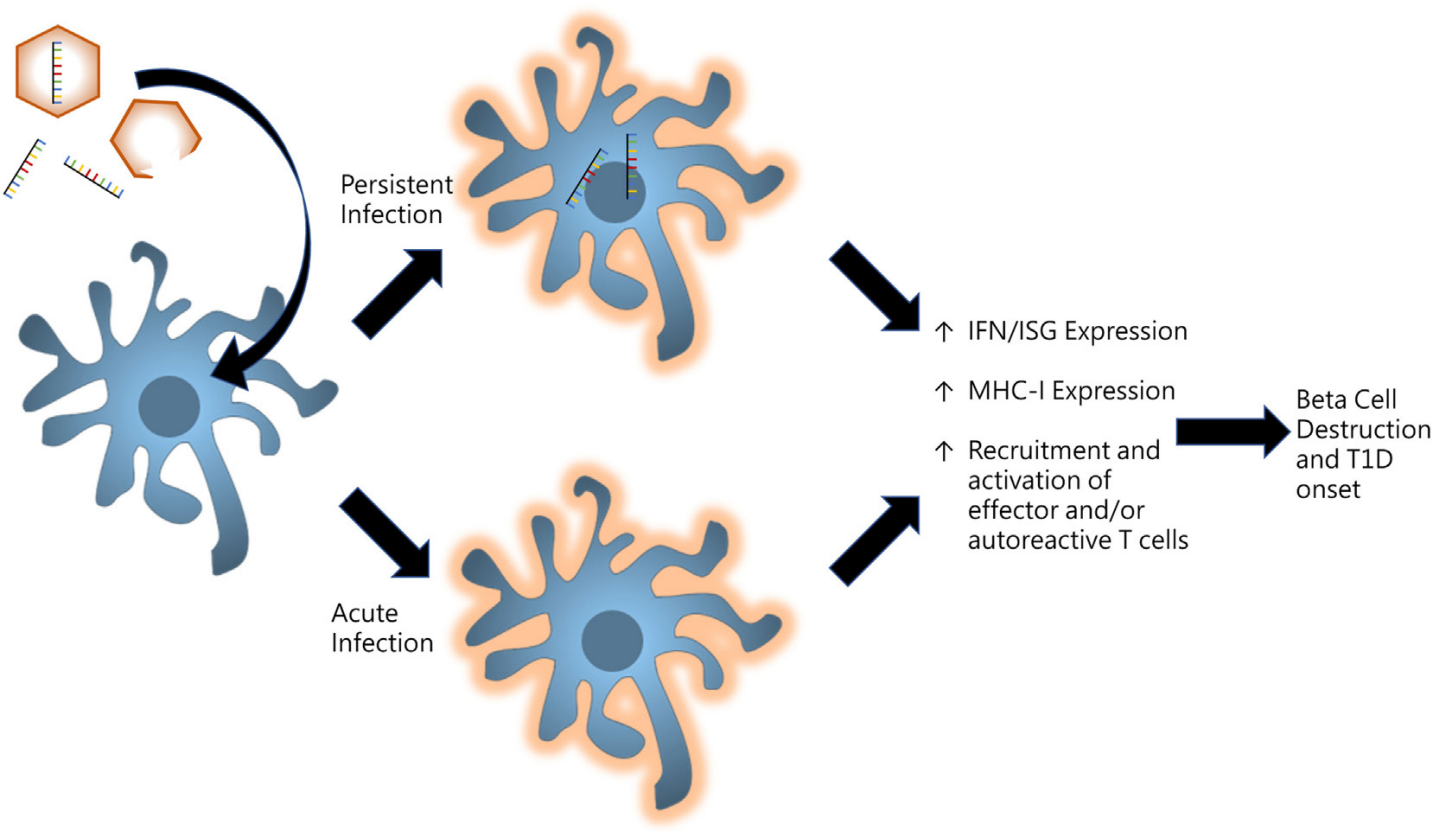
Model for persistent versus acute infections in pancreas for promoting type I diabetes (T1D). Virus infections induce proinflammatory signaling in resident and infiltrating antigen-presenting cells of the pancreas. Persistent infections whereby viral products including enteroviral 5′ terminally deleted double-stranded RNA are maintained in tissue microenvironments to produce low-grade inflammatory signaling. Alternatively, acute infections may result in unregulated positive feedback mechanisms which produce chronic inflammation even after the virus is cleared.

With regards to T1D induction, it is unclear whether virus infections are being sustained or acute infections are initiating mechanisms which go on unregulated even after viral clearance. If the virus becomes persistent, remnants of the infection linger within tissue-specific microenvironments to provide continuous stimulation of innate receptors to produce chronic inflammation as illustrated in Figure 2. Some picornaviruses such as Theiler’s murine encephalomyelitis virus have been shown to persist in certain tissues to provide sufficient inflammation to drive autoimmunity (84). Conversely, acute infections may be priming the host and establishing events which direct an autoreactive effector response. Initial infections may be activating pathways which proceed with incessant positive feedback likely due to genetic differences which result in functional variations in innate compsonents, receptor activation/deactivation, and/or signaling pathway elements.

Coxsackievirus B is a positive sense ssRNA virus with a tropism for the pancreas, heart, and liver; however, it does not appear to establish cytolytic infections of pancreatic beta cells (85). Rather, CVB is likely promoting a pro-inflammatory environment within the pancreas to elicit autoimmunity (86). Infecting human islets with CVB3 induces potent expression of type I and III IFN, MDA5, RIG-I, and TLR3 along with a variety of inflammatory cytokines (32). Clinical evidence suggests some individuals who develop a loss of tolerance against insulin early in life (1–3 years) have an impaired capacity to mount a sufficient defense against the enterovirus viral capsid protein, viral protein 1 (VP1), that may cause an inability to sufficiently clear CVB following infection (87). Correspondingly, persistent pancreatic cell CVB4 infections have been shown to induce epigenetic changes by promoting production of dysregulated microRNAs targeting T1D risk genes (88).

Prolonged viral infections may be providing sustained activation of PRR signaling for the expression of IFN-I, IFN-III, and inflammatory cytokines, leading to a robust lymphocyte response and induction of autoimmunity. Low-grade enterovirus infections have been demonstrated to be established and maintained within the pancreatic islets of patients recently experiencing onset of T1D (3–9 weeks prior) but rarely in healthy controls (89). Persistent enteroviral presence has also been detected in the gut mucosa of T1D patients, however, viral genomes seem to be maintained in the absence of viral protein expression or production of infective particles (90). It is not clear whether defective replication is allowing production of the viral genes without assembly of virus particles or if virus components are simply persisting in the tissue after infections. However, conformational differences and modification of viral PAMPS may be dictating innate signaling. Stem-loop structures in long dsRNA are preferentially recognized by MDA5, while 5′ di- or triphosphate motifs on dsRNA are sensed by RIG-I (28, 91). Deletion or alterations in the structural composition of viral genomic PAMPs may be altering innate receptor signaling. CVB is known to persist in myocardial tissues following naturally occurring deletion of 5′ end terminal genomic sequences resulting in reduced virus replication and loss of cytopathicity (92–94). A recent study has shown that 5′ terminally deficient CVB persists also in the pancreas of NOD mice for at least several weeks following an acute wave of infection (95). The 5′ end of the CVB genome includes a cloverleaf-like tertiary structure which may be favorably sensed by MDA5; however, it is unknown exactly how innate sensing is affected with these terminal deleted viruses. Persistence of modulated enteroviruses may be providing sustained innate activation for prolonged inflammatory responses in and/or around the islets that result in the loss of self-tolerance in T1D. Alternatively, loss of structurally relevant motifs for RLR sensing may actually render receptors like MDA5 unable to bind the modified dsRNA ligands.

### Temporal Determinants of T1D-Related Virus Infections

A systematic review compiling and analyzing clinical studies over approximately the last two decades, found that individuals had about 10 times higher odds of having enterovirus infections before or during onset of diabetes or prediabetes when compared to controls (24). Patients experiencing fulminant T1D directly following suspected enterovirus infection had strong expression of MDA, RIG-I, and VP1 in the islets when compared to T1D and non-diabetic control patients (96). Furthermore, mononuclear cells which infiltrate the pancreata of patients experiencing fulminant T1D had high expression of TLR3 and TLR4 (96). A recent study by Laitinen et al. screening systematically collected blood samples from birth through seroconversion for T1D-related autoantibodies and progression to clinical T1D, found that children were at higher T1D risk if infected with CVB1 (97). However, the patient was protected if exposed to either CVB3 or CVB6 prior to CVB1. Phylogenetic similarity between CVB1, CVB3, and CVB6 indicates the possibility that cross-protection between highly related enterovirus serotypes may be occurring. Additionally, CVB1 infection has been often followed by the appearance of islet autoantibodies about 6 months later (98, 99).

Characterizing temporal relationships between infection and autoimmunity onset are incredibly intricate due to the incredibly multifactorial nature of the pathogen, the hosts, and the disease. The timing of pathogen exposure and an individual’s age likely has crucial impact on immunological development (100). It was recently determined that weaning pups from a colony of NOD mice with low incidence of T1D in a “diabetogenic environment” (i.e., with a colony of NOD mice with high T1D incidence) is able to transfer rates of diabetes development by adapting similar gut microbiota and promoting development of B cells in the mesenteric lymph nodes which are inherently more easily activated (101). This transmittance is only evident when the mice are weaned together, as this environmental exposure does not affect rate of diabetes onset when mice are co-housed starting at 3 weeks of age. Mustonen et al. performed a clinical analysis among children with HLA-dependent T1D genetic risk (exhibited DR3-DQ2 and/or DR4-DQ8 haplotypes) in Finland, Estonia, and Russian Karelia to determine disease trends in T1D susceptibility (102). Children who exhibited seroconversion of T1D-related autoantibodies had their first infection earlier and overall had more infections in the first year of their lives especially in the respiratory tract. Furthermore, those which progressed to T1D had twice as many infections in the first 3 years of their lives than non-diabetic children. It can be questioned, however, whether inherent susceptibility to T1D also confers lower tolerance to pathogens or whether the children experienced onset of T1D due to stress of the frequency of infections they experienced. A report from The Environmental Determinants of Diabetes in the Young study has confirmed that young children experiencing recent respiratory infections withstand a heightened risk of developing T1D-related autoimmunity; however, more work is necessary to determine specific viral agents present in the preceding months before autoantibody seroconversion (103).

While enteroviruses remain the most likely candidate for T1D onset, numerous other viruses have been shown to have roles in promoting or protecting T1D (104). Links between many viruses, however, seem to be more circumstantial and less evident. For instance, a study was performed examining the spatio-temporal exposure of viruses using geographical disease incidence rates in France and relating that data with mapping of T1D patient residences and the timing of the patients’ T1D onset (105). This analysis indicates a positive correlation between summer diarrhea and influenza-like infections at 1–3 years of age with eventual development of T1D while there was negative relationship between varicella. Additionally, evidence suggests autoreactivity in NOD mice may be induced as a consequence of an immunological response against endogenous retrovirus-secreted microvesicles in the islets (106). Recently, a study using high-throughput proteomic profiling of antibodies in new-onset T1D patients found serum antibodies exhibit a significant reactivity against Epstein–Barr virus viral antigens (107). Ultimately, a multifactorial and heterogenous contribution from multiple environmental agents is likely for T1D pathogenesis.

## Conclusion

The increased incidence of autoimmunity witnessed in developed nations likely signifies a deleterious shift in pathogenic environment especially early in life. This may be due to modern alterations in the host–pathogen paradigm developed over milleniums of co-evolvement. Epidemiological studies have not indicated an emergence of infections that could adequately explain such a significant increase in autoimmunity. Thus, it is likely caused by an alteration in how individuals respond to environmental and pathogenic stressors. One rationalization for this change, the “hygiene hypothesis,” states that a reduction in pathogenic and environmental antigen exposure particularly during development has caused the immune system to produce over-exaggerated responses resulting in increased rates of autoimmunity. Decreased interaction with typical environmental antigens has fostered inexperience by innate host receptors, causing over-sensitization and improper stimulation of inflammatory pathways.

Environmental induction of T1D *via* viral infection may essentially require a “perfect storm” of immune reactivity where genetic susceptibility allows PRR signaling to render a target organ susceptible to attack by self-reactive lymphocytes. A balance of signaling by different receptors including RLRs and TLRs is providing opposing forces to simultaneously promote and inhibit autoimmunity and certain environmental stressors may be sufficient to tip that balance toward autoimmunity by inducing pro-inflammatory signaling. Ultimately, the timing, pathogenesis, and target site of infection influences the likelihood of antigen-non-specific bystander activation of autoreactive B and T cells. Understanding these pathways may hold a high degree of therapeutic potential to block onset of autoimmunity by mediating antigen exposure, developing relevant vaccines, and managing molecular pathogenesis mechanisms which confer disease development.

## Author Contributions

ZM and MH conceptualized, wrote, and edited the manuscript. ZM created the figures.

## Conflict of Interest Statement

The authors declare that the research was conducted in the absence of any commercial or financial relationships that could be construed as a potential conflict of interest.

## Abbreviations

CVB, coxsackievirus B; DC, dendritic cell; dsRNA, double-stranded RNA; GWAS, genome-wide association studies; IFIH1, interferon induced with helicase C domain 1; IFN, interferon; IFNAR, type I interferon receptor; IFNλR, type III interferon receptor; IRF7, interferon regulatory factor 7; LCMV, lymphocytic choriomeningitis virus; MDA5, melanoma differentiation-associated protein 5; MYD88, myeloid differentiation primary response gene 88; NOD mice, non-obese diabetic mice; pDC, plasmacytoid dendritic cell; poly I:C, polyinosinic:polycytidylic acid; PRR, pattern-recognition receptor; RA, rheumatoid arthritis; RIG-I, retinoic acid-inducible gene I; RLR, RIG-I-like receptors; SLE, systemic lupus erythematosus; SNP, single nucleotide polymorphism; ssRNA, single-stranded RNA; T1D, type 1 diabetes; TLR, toll-like receptor; T_REG_, regulatory T cell; VP1, viral protein 1.
